# Risk Factors for Bacterial Contamination of Bovine Meat during Slaughter in Ten Indonesian Abattoirs

**DOI:** 10.1155/2019/2707064

**Published:** 2019-11-19

**Authors:** Diyantoro, Dhandy Koesoemo Wardhana

**Affiliations:** ^1^Department of Health Science, Faculty of Vocational Studies, Universitas Airlangga, Surabaya, Indonesia; ^2^Department of Veterinary Public Health, Faculty of Veterinary Medicine, Universitas Airlangga, Surabaya, Indonesia

## Abstract

Provision of beef meat which does not exceed the maximum microbial contamination limit is expected to meet the requirements to obtain safe, healthy, wholesome, and halal beef. Bacterial contamination during slaughtering process is a safety problem and concern for shelf life in meat production. This study was designed to determine the value of microbial contamination and its risk factors at the stage of the slaughtering process in the abattoirs. This research was conducted by visual observation accompanied by questionnaires and laboratory examination for bacterial contamination testing. The results showed the factor that significantly affected the total plate count (TPC) was carcass cutting (mean: 0.46 × 10^6^ CFU/g; *p* = 0.035) which was not carried out by the abattoir. The factor that had the greatest effect on the MPN of *Escherichia coli* was blood removal on the floor position (mean: 40.34 × 10^6^ CFU/g; *p* = 0.039) while the factors that significantly affected *Staphylococcus aureus* contamination were blood removal on the floor position (mean: 52.88 × 10^6^ CFU/g; *p* = 0.025) and carcass cutting which were not carried out by the abattoir (mean: 66.42 × 10^6^ CFU/g; *p* = 0.015).

## 1. Introduction

Food of animal origin should be monitored to ensure that people can obtain consumable meat. Beef meat may include biological, physical, and chemical hazards that may occur at any point during the supply process from slaughtering to table. Pathogenic microorganisms are normally found in the digestive tract of healthy cattle. These microorganisms can also be found on the hides of live animals contaminated from feces which can then be transferred to the surface of previously sterile meat during slaughtering especially when performed on the floor with the absence of a carcass suspension system with careless evisceration that spreads intestinal content onto the meat surface. Bovine carcasses can be contaminated during the slaughter process through contact with the animal's skin and hair, limbs, blood, stomach, gut contents, bile, and other excretions, facilities, equipment, and hands and worker's clothes [1]. For those reasons, special attention is needed in the implementation of hygiene and sanitation during the slaughtering process. The important steps preparing for the quality and safe meat occur in abattoirs. Abattoirs are a community service unit that are intended to provide safe, healthy, and wholesome halal meat, a place for hygienic slaughter, and a place for monitoring and surveillance of animal diseases and zoonoses [2].

According to the Directorate General of Animal Husbandry and Animal Health, Ministry of Agriculture Republic Indonesia [3], there are only 25 abattoirs of around 800 abattoirs in Indonesia that have a veterinary certificate, which are official and legal benchmarks indicating hygiene-sanitation requirements as basic feasibility guarantee of food safety from animals produced by the abattoir. Article 62 of Law 18/2009 concerning animal husbandry and animal health states that the district or city government must have an abattoir that meets technical requirements. From this statement, it is clear that the law mandates regional governments to fulfill the technical requirements of abattoirs in their territory. However, in reality, the abattoir has the main function for providing consumers with halal slaughtered livestock, safe meat and maintaining the quality of produced-meat, although at present this requirement is not always fulfilled. The supply of beef meat which does not exceed the maximum microbial contamination limit is expected to meet the above requirements to provide safe, healthy, wholesome halal beef. An abattoir is not a sterile environment and has a high risk of pathogenic microbial contamination. After the cattles are processed, the microflora found in animals begin to invade the tissue so that the meat will spoil quickly if the product is not handled correctly [4].

The previous study reported that several meat samples from city slaughterhouses in East Java, Indonesia was found to have microbial contamination including *Escherichia coli* (32.5%), *Staphylococcus aureus* (20%), and *Salmonella* sp. (2.5%) [5]. In addition to the highest prevalence rate, the mean value of *Escherichia coli* contamination was higher than the maximum limit of microbial contamination based on the National Standardization Agency of Indonesia [6]. Good Slaughtering Practices (GSP) includes all practices in abattoir relating to the conditions and actions needed to ensure the safety of food at all stages in the food chain [7]. Harris and Jeff [8] stated that the implementation of GSP serves to minimize the contamination of diseases from preslaughter, handling the livestock in the lairages washing, stunning, slaughtering, and carcass washing. In addition, the GSP stages should also include the cleanliness of production facilities, water used during the process, implementation of sanitation programs, and validation processes. This study was aimed to determine the value of microbial contamination including total plate count (TPC), *Staphylococcus aureus*, and *Escherichia coli* and its risk factors at the stage of the slaughtering process in abattoirs.

## 2. Materials and Methods

### 2.1. Abattoirs Selection

Ten city abattoirs in East Java Province were selected in this study including Kedurus Abattoir and Pegirian Abattoir located in Surabaya City, Mojokerto City Abattoir, Pasuruan City Abattoir, Gadang Abattoir located in Malang City, Batu City Abattoir, Kediri City Abattoir, Probolinggo City Abattoir, Dimoro Abattoir located in Blitar City, and Madiun City Abattoir.

### 2.2. Observation and Data Collection

General characteristics and slaughtering processes were observed visually from each abattoir. The slaughtering process, such as slaughtering, blood removal techniques, meat cutting, rigor mortis process, skin preparation and evisceration process, and subcutaneous fat trimming, were carefully perceived.

### 2.3. Meat Sample Collection

Four samples from each abattoir were collected from ten city abattoirs. The samples were collected with an attempt to minimize microbial contamination caused by environmental temperatures; hence it was done in the early morning and within 8 hours postslaughter. One hundred grams of raw beef meat (gluteus medius) samples were collected from each sample. Then ten grams of the collected meat sample were further transfered to sterile flask containing 90 ml of distilled water. Under aseptic condition, the samples were homogenized using the pestle and mortar.

### 2.4. Microbiological Examination

#### 2.4.1. Total Plate Count (TPC)

Total Plate Count was performed initially by homogenized 25 grams of each meat sample with 225 ml of 1% Buffered Peptone Water (BPW) (Merck 1.07228.0500) for 1-2 minutes then put it into serial dilution. Five sterile test tubes were labeled as 10^−1^ to 10^−5^for serial dilution. One ml of diluted meat sample was mixed thoroughly with nine ml of BPW in the first test tube and labeled as 10^−1^. One ml solution was taken from the first test tube and transferred to the second test tube labeled as 10^−2^. This was continued until 10^−5^ dilution was obtained. Then one ml of meat samples from each dilution was inoculated on Nutrient Agar (NA) (Merck 1.05450.0500) plates, and then incubated at 37°C for 18−24 hours. After 24 hours, the observed growing colonies from the NA plates were counted for *Total Plate Count* (TPC) [9].

#### 2.4.2. Most Probable Number (MPN) *Escherichia coli*

The viable numbers of *Escherichia coli* in a sample were estimated using the MPN method. One ml sample was added into 9 ml BPW media then a serial dilution of three test tubes were set as 10^−1^ to 10^−3^. One ml of each diluted samples were transferred into five tubes containing brilliant green bile broth (BGBB) media (Merck 1.05454.0500) with inserted Durham tube and incubated at 45.5°C for 24−48 hours. *Escherichia coli* were observed in those tubes which produced gas. The confirmation test was performed by inoculating 1 loop of positive *Escherichia coli*-containing broth into an Eosin Methylene Blue Agar (EMBA) (Merck 1.01347.0500). Green metallic appearance will be observed for sample containing *Escherichia coli*. These samples then further proceeded for indol testing and transferred into tryptone water media (Merck 1.10859.0500). The MPN value was calculated based on the number of tubes with positive *Escherichia coli* broth dilution using McGrady's table [10].

#### 2.4.3. Detection of *Staphylococcus aureus*

*Staphylococcus aureus* was detected with inoculation of 0.1 ml from the first dilution (10^−1^) into Mannitol Salt Agar (MSA) (Merck 1.05404.0500) after 24 hours at 37°C. *Staphylococcus aureus* gave yellow colony appearance on MSA while other species of *Staphyloccocus* showed red colonies [11].

### 2.5. Data Analysis

Data from visual observation in the field and laboratory tests is analyzed by one-way analysis of variance (ANOVA) using SPSS [12] statistical software (Ver.16.0 for windows, SPSS Inc, Chicago, IL, USA) to determine the significance (*p*-value) of the mean values of each Total Plate Count, *Staphylococcus aureus*, and *Escherichia coli* tests between categories of each parameter was tested. In addition, a regression analysis test was also conducted to determine the percentage of positive sample and Odd Ratio (OR) value of each parameter for determining the risk factors or exposure associations of microbial contamination.

## 3. Results and Discussion

The general information of the ten selected abattoirs in Table 1 showed that most abattoirs were owned by service technical implementation units. Meanwhile, based on the slaughtering process stages that were known, it was shown that 90% of slaughtering activities were carried out by the abattoir, 60% of blood removal was performed by hanging position called vertical bleeding, 70% of the carcass cutting was carried out by the abattoir and 60% of the carcass was cut into quarters, the rigor mortis did not occur in 90% of the abattoirs, 70% of the evisceration and skinning process were performed on the floor while 60% of abattoirs performed subcutaneous fat trimming.

The spread of microorganisms that grow in food from animals and their processed products in general consists of bacteria, fungi/molds, viruses along with unicellular organisms, and there are also one cell animals. Meat serves as a food to many microorganisms and similarly can be contaminated by these microorganisms. Microbial contamination in meat can start from the first skin incision made to remove the blood, especially if the tools and equipment used by the operator are not sterile. Subsequent contamination can occur on the surface of the meat during meat preparation, carcass or meat cutting, manufacturing of processed meat products, packing, storage, and distribution. So, anything that can contact meat directly or indirectly, can be a source of microbial contamination [13].

Based on the TPC result seen in Table 2, the highest positive percentage was found at the stage with rigor mortis during slaughtering process (25%), but the highest mean value of TPC was found in the process of carcass cutting which was not carried out by abattoir (0.46 × 10^6^ CFU/g) and was the only one that had a very significant influence on the TPC value (*p* = 0.035). An increase in pH after rigor mortis induces bacterial growth of putrefactive bacteria that produces proteinase which causes spoilage [14]. The spoilage of meat depends on storage temperature, biodiversity of bacterial groups, availability of oxygen, and pH level [15].

Table 3 showed that the only factor having a statistically significant effect on MPN of *Escherichia coli* was blood removal performed on the floor with a positive percentage of 45.8%, the mean value of 40.34 × 10^6^ CFU/g, and *p-value* of 0.039.

There were two stages of the slaughtering process that had a significant effect on the value of *Staphylococcus aureus* contamination including blood removal performed on the floor (*p* = 0.025) and carcass cutting which was not carried out by abattoir (*p* = 0.015). The percentage of positive samples of *Staphylococcus aureus* contamination in the blood removal stage was 25% with the mean value of 52.88 × 10^6^ CFU/g, while in the carcass cutting stage was 33.3% with the mean value of 66.42 × 10^6^ CFU/g (Table 4).

Blood removal performed on the floor is very unhygienic. The process should take place on a clean stainless-steel table which should be cleaned frequently. One of the main sources of contamination during bleeding is knife. The knife should be changed after operation and returned to a sterilizer [16]. Bleeding and skinning of neck, cheeks, shoulder, and legs were done on the floor and contamination from the hide of one animal to others was transmissible. In the cattle slaughter line, all the slaughtering processes were performed on a production line with vertical rail dressing and automatic hide pullers. Hygienic condition of bleeding in cattle slaughter line was better when the animals hoisted by one leg and bleeding continues until the blood flow is negligible. In general, contamination of carcasses is reduced by using automatic hide removal because there is less handling of the carcass and less use of knives. Vertical rail dressing improves hygienic practice by reducing carcass contact with operators, equipment, and other carcasses [17].

The cutting of carcasses, involves the use of utensils, equipment and knives and may allow for the transfer of more microorganism to beef tissues. Furthermore, workers' hands, clothes and their instruments may spread contamination onto the surface of beef carcasses [18]. In most developing countries, the absence or poor hygienic practices in slaughtering, dressing and evisceration has been found to be one of the major causes of high surface contamination of beef carcasses by pathogenic and nonpathogenic microorganism [19].

One of the main sources of higher values of staphylococcal counts on the surface of examined carcasses are abattoir workers, as their hands were found to be highly contaminated; this is in accordance with the reports of Schlegelova et al. [20]. Additionally, Dickson and Anderson [21] and Sokari and Anozie [22] mentioned that contaminated skin, faeces, the contents of digestive organs, butchers' knives, hands, clothes, and contaminated water are the main sources of contamination with Staphylococcal spp. during meat processing.

The surface contamination of beef carcasses with coliforms could be attributed to contamination from their intestine; however, hides and hooves contain a large number of such organisms from soil, manure, and feed and may be transferred to the carcass during dressing. Besides, the contaminated water, utensils, and equipments used in carcass slaughtering, dressing and evisceration, these results support the views previously reported of Guthrie [23]; Gracey and Collins [18] and Marriot [24].

## 4. Conclusions

There were several stages of the slaughtering process that had significant effects on microbial contamination. The only factor that had a significant effect on the TPC value was carcass cutting which was not carried out by an abattoir, while with regard to the MPN value, the only significant value related to blood removal performed on the floor. There were two stages of the slaughtering process that had a significant effect on the value of *Staphylococcus aureus* contamination and again these included blood removal performed on the floor and carcass cutting which was not carried out by the abattoir.

## Figures and Tables

**Table 1:** General information of ten selected abattoirs.

Information	Category	Number of observed (*n*)	Proportion (%)
Ownership status	Regional owned enterprises	3	30
	Service technical implementation unit	7	70
Slaughtering	Carried out by abattoir	9	90
	Not carried out	1	10
Blood removal techniques	Hanging position	4	40
	On the floor position	6	60
Carcass cutting	Carried out by abattoir	7	70
	Not carried out	3	30
	Cut into quarters	6	60
	Cut into halves	2	20
	Cut into other sizes	2	20
Rigor mortis process	Yes	1	10
	No	9	90
Skin preparation and evisceration process	Hanging position	3	30
	On the floor position	7	70
Subcutaneous fat trimming	Yes	6	60
	No	4	40

**Table 2:** Total plate count.

Slaughtering Process Stage	Category	% pos.	OR	*p* value	Mean (10^6^ CFU/g)	CI	*p* value
Blood Removal Techniques	Hanging position	6.3 (1/16)	1.5	0.769	0.16	(0.03)–0.36	0.206
	On the floor position	4.2 (1/24)	Ref.		0.33	0.15–0.50	
Carcass cutting	Not carried out	8.3 (1/12)	0.4	0.538	0.46	0.13–0.79	**0.035 ** ^∗^
	Carried out by abattoir	3.6 (1/28)	Ref.		0.18	0.06–0.29	
	Cut into quarters	12.5 (1/8)	7.0	0.999	0.38	(0.14)–0.89	0.410
	Cut into halves	4.2 (1/24)	2.3	0.999	0.27	0.12–0.43	
	Cut into other sizes	0.0 (0/8)	Ref.		0.11	0.01–0.22	
Rigor mortis process	Yes	25.0 (1/4)	11.7	0.110	0.36	(0.73)–1.44	0.624
	No	2.8 (1/36)	Ref.		0.25	0.13–0.37	
Skin separation and Eviseration process	Hanging position	8.3 (1/12)	2.5	0.538	0.27	(0.06)–0.59	0.954
	On the floor position	3.6 (1/28)	Ref		0.26	0.13–0.39	
Subcutaneous Fat Trimming	Yes	8.3 (2/24)	1.5	0.999	0.34	0.14–0.54	0.132
	No	0.0 (0/16)	Ref		0.15	0.05–0.25	

^∗^Indicates a significant difference of *p* < 0.05.

**Table 3:** MPN *Escherichia coli*.

Slaughtering process stage	Category	% pos.	OR	*p* value	Mean (10^6^ CFU/g)	CI	*p* value
Blood removal techniques	On the floor position	45.8 (11/24)	0.2	**0.039^∗^**	40.34	7.56–73.13	0.097
	Hanging position	12.5 (2/16)	Ref.		7.05	3.43–10.67	
Carcass cutting	Not carried out	50.0 (6/12)	0.3	0.129	32.25	(9.95–74.25)	0.737
	Carried out by abattoir	25.0 (7/28)	Ref.		24.83	1.09–48.57	
	Cut into quarters	50.0 (4/8)	3.5	0.277	41.73	(25.82–109.27)	0.534
	Cut into halves	33.3 (8/24)	7.0	0.129	28.78	1.09–54.48	
	Cut into other sizes	12.5 (1/8)	Ref.		7.05	1.12–46.86	
Rigor mortis process	Yes	50.0 (2/4)	2.3	0.440	14.55	(1.49)–30.59	0.667
	No	30.6 (11/36)	Ref.		28.41	6.34–50.48	
Skin separation and eviseration process	On the floor position	39.3 (11/28)	0.3	0.175	35.42	7.24–63.62	0.194
	Hanging position	16.7 (2/12)	Ref.		7.42	2.56–12.27	
Subcutaneous fat trimming	Yes	33.3 (8/24)	0.9	0.890	20.28	0.14–40.45	0.406
	No	31.3 (5/16)	Ref.		37.15	(5.25)–79.55	

^∗^Indicates a significant difference of *p* < 0.05.

**Table 4:** *Staphylococcus aureus*.

Slaughtering process stage	Category	% pos.	OR	*p* value	Mean (CFU/g)	CI	*p* value
Blood removal techniques	On the floor position	25.0 (6/24)	0.2	0.156	52.88	31.54–74.21	**0.025^∗^**
	Hanging position	6.3 (1/16)	Ref.		19.56	2.49–36.63	
Carcass cutting	Not carried out	33.3 (4/12)	0.24	0.099	66.42	30.00–102.83	**0.015^∗^**
	Carried out by abattoir	10.7 (3/28)	Ref.		28.04	13.83–42.24	
	Cut into quarters	62.5 (5/8)	1.0	1.000	55.75	3.04–108.46	0.532
	Cut into other sizes	25.0 (2/8)	0.4	0.408	31.00	(9.05)–71.05	
	Cut into halves	0.0 (0/24)	Ref.		37.00	19.83–54.17	
Rigor mortis process	No	19.4 (7/36)	0.0	0.999	41.58	25.42–57.75	0.415
	Yes	0.0 (0/4)	Ref.		21.25	(32.01)–74.51	
Skin separation and eviseration process	On the floor position	17.9 (5/28)	0.9	0.928	40.29	23.74–56.83	
	Hanging position	16.7 (2/12)	Ref.		37.83	1.72–73.95	0.881
Subcutaneous fat trimming	Yes	20.8 (5/24)	1.8	0.501	46.25	24.82–67.68	0.271
	No	12.5 (2/16)	Ref.		29.50	8.72–50.28	

^∗^Indicates a significant difference of *p* < 0.05.

## Data Availability

The data used to support the findings of this study are included within the article.

## References

[1] Sofos J. N. (2008). Challenges to meat safety in the 21^st^ century. *Meat Science*.

[2] Tawaf R. (2013). Physical and technical feasibility procedure slaughter at the Government Abattoir in West Java. *Seminar Nasional Peternakan Berkelanjutan ke 5 Fapet Unpad*.

[3] Directorate General of Livestock and Animal Health (2012). *Realizing the Indonesia Slaughtering Management that has the Principle of Animal Welfare*.

[4] Rahayu E. S. (2006). Are our food products secure: freedom from dangerous contamination. The appreciation for quality improvement of agricultural processed products.

[5] Soepranianondo K., Wardhana D. K., Budiarto, Diyantoro (2019). Analysis of bacterial contamination and antibiotic residue of beet meat from city slaughterhouses in East Java Province, Indonesia. *Veterinary World*.

[6] The National Standardization Agency of Indonesia (2008). *Indonesian National Standard Number 7424: 2008 About the Screening Test Method of Antibiotic Residues on Meat, Eggs, and Milk by Bioassay*.

[7] Codex Alimentarius Commission (2004). *Join FAO/WHO Food Standard Programme. Report of the Tenth Session of the Codex Committee on Meat Hygiene*.

[8] Harris K. B., Jeff W. S. (2003). *Best Practices for Beef Slaughter*.

[9] Brown A. E. (2009). *Benson’s Microbiological Application: Laboratory Manual in General Microbiology*.

[10] Prawesthirini S., Siswanto H. P., Estoepangestie A. T. S. (2016). *Analysis of Meat, Milk, and Egg Quality*.

[11] Jawetz E., Melnick J. L., Adelberg E. A. (2007). *Medical Microbiology*.

[12] SPSS (2008). *SPSS 16.0 for Windows*.

[13] Soeparno (2009). *Meat Science and Technology*.

[14] Nowak A., Rygala A., Oltuszak-Walczak E. P., Walczak P. (2012). The prevalence and some metabolic traits of Brochothrix thermosphacta in meat and meat products packaged in different ways. *Journal of the Science of Food and Agriculture*.

[15] Ercolini D., Russo F., Nasi A., Ferranti P., Villani F. (2009). Mesophilic and psychrotrophic bacteria from meat and their spoilage potential in vitro and in beef. *Applied and Environmental Microbiology*.

[16] FAO (1991). *Guideline for slaughtering, meat cutting, and further processing. Animal production and health paper*.

[17] Bakthtiary F., Sayevandi H. R., Remely M., Hippe B., Hosseini H., Halsberger A. G. (2016). Evaluation of bacterial contamination sources in meat production line. *Journal of food quality*.

[18] Gracey J. F., Collins D. S. (1992). *Meat Hygiene*.

[19] Eugène N., Divine B., Martin P. O. (2013). Assessment of beef meat microbial contamination during skinning, dressing, transportation and marketing at a commercial abattoir in Kigali city, Rwanda. *Pakistan Journal of Food Sciences*.

[20] Schlegelova J., Napravnikova E., Dendis M. (2004). Beef carcass contamination in a slaughterhouse and prevalence of resistance to antimicrobial drugs in isolates of selected microbial species. *Meat Science*.

[21] Dickson J. S., Anderson M. E. (1992). Microbiological decontamination of food animal carcasses by washing and sanitizing systems: a review. *Journal of Food Protection*.

[22] Sokari T. G., Anozie S. O. (1990). Occurrence of enterotoxin producing strains of *Staphylococcus aureus* in meat and related samples from traditional markets in Nigeria. *Journal of Food Protection*.

[23] Guthrie R. K. (1988). *Food Sanitation*.

[24] Marriot N. G. (1997). *Essentials of Food Sanitation*.

